# Analysis of In Vitro Leukocyte Responses to Biomaterials in the Presence of Antimicrobial Porcine Neutrophil Extract (AMPNE)

**DOI:** 10.3390/ma16165691

**Published:** 2023-08-19

**Authors:** Beata Drzewiecka, Agata Przekora, Dominika Dobko, Aleksandra Kozera, Katarzyna Krać, Dominika Nguyen Ngoc, Eric Fernández-De la Cruz, Joanna Wessely-Szponder

**Affiliations:** 1 Sub-Department of Pathophysiology, Department of Preclinical Veterinary Sciences, Faculty of Veterinary Medicine, University of Life Sciences, Akademicka 12, 20-033 Lublin, Poland; beata.drzewiecka@up.lublin.pl (B.D.); dominika.nguyen@up.lublin.pl (D.N.N.); 2 Independent Unit of Tissue Engineering and Regenerative Medicine, Medical University of Lublin, Chodzki 1 Street, 20-093 Lublin, Poland; 3 Students Research Group of Veterinary Analysts, Sub-Department of Pathophysiology, Department of Preclinical Veterinary Sciences, Faculty of Veterinary Medicine, University of Life Sciences, Akademicka 12, 20-033 Lublin, Poland; dobkodominika@gmail.com (D.D.); aleksandra1.kozera@gmail.com (A.K.); kasiakrac@gmail.com (K.K.); 4 Department of Pathology & Experimental Therapeutics, Faculty of Medicine & Health Sciences, University of Barcelona, 08907 Barcelona, Spain; eric.fernandez@ub.edu

**Keywords:** antimicrobial peptides, biomaterial, blood derived products, inflammation, macrophages, neutrophils

## Abstract

Implant insertion can evoke excessive inflammation which disrupts the healing process and potentially leads to complications such as implant rejection. Neutrophils and macrophages play a vital role in the early inflammatory phase of tissue repair, necessitating the study of cellular responses in host–implant interactions. In order to deepen the knowledge about these interactions, the response of neutrophils and macrophages to contact with selected biomaterials was examined in vitro on the basis of secretory response as well as reactive oxygen species/reactive nitrogen species (ROS/RNS) generation. Porcine neutrophils exposed to hydroxyapatite (HA) released more enzymes and generated higher levels of ROS/RNS compared to the control group. The addition of AMPNE diminished these responses. Although the results from porcine cells can provide valuable preliminary data, further validation using human cells or clinical studies would be necessary to fully extrapolate the findings to human medicine. Our study revealed that human neutrophils after contact of with HA increased the production of nitric oxide (NO) (10.00 ± 0.08 vs. control group 3.0 ± 0.11 µM, *p* < 0.05), while HAP or FAP did not elicit a significant response. Human macrophages cultured with HA produced more superoxide and NO, while HAP or FAP had a minimal effect, and curdlan reduced ROS/RNS generation. The addition of AMPNE to cultures with all biomaterials, except curdlan, reduced neutrophil activity, regardless of the peptides’ origin. These results highlight the potential of antimicrobial peptides in modulating excessive biomaterial/host cell reactions involving neutrophils and macrophages, enhancing our understanding of immune reactions, and suggesting that AMPNE could regulate leukocyte response during implantation.

## 1. Introduction

Excessive inflammation may disturb healing after biomaterial implantation and could be the source of serious complications all the way to implant rejection. Cells involved in the immune response can influence the course and outcome of the tissue repair process. Neutrophils are the first cells responding to the implantation of biomaterials during the immunoinflammation phase of tissue repair. These cells migrate to the focus of injury within hours and contribute to scavenging the damaged tissues and debris through phagocytosis, releasing enzymes and generating reactive oxygen/nitrogen species (ROS/RNS). In the further stages of repair process, they are replaced by monocytes and monocyte-derived macrophages (MDM). Macrophage activity, in turn, may be modified by interactions with biomaterials to enhance healing and material integration [1]. Generally, the main participants of this process are the blood-derived cells migrating to the site of injury. Therefore, the modulation of this early phase of repair process has drawn attention to enhance the tissue regeneration properties of biomaterials [2]. For these reasons, we decided to evaluate the activity of some inflammatory cells crucial for the immune response to implanted biomaterial, namely neutrophils and macrophages. For this purpose, we assessed the ROS/RNS generation of both subpopulations of leukocytes and additionally the secretory response of neutrophils based on enzyme release. Hydroxyapatite (HA) is a kind of calcium phosphate ceramic with high biocompatibility, osteoinductivity and the ability to osseointegration. It has been developed as an implantable material with potential for bone regeneration in different forms such as bone substitutes, scaffolds and implant coatings and is widely used in orthopedics and dentistry. However, HA has the tendency toward the fragmentation and release of small particles, which can lead to neutrophil and monocytic cell activation during repair process, which can cause implantation failure [1,3]. While mechanical parameters and the stability of HA are usually improved via the production of composite biomaterials, its biological properties may be significantly modified with various ionic substitutions, e.g., magnesium (Mg^2+^), silicon (Si^2+^), zinc (Zn^2+^), copper (Cu^2+^), strontium (Sr^2+^), cobalt (Co^2+^), lithium (Li^+^), or fluoride (F^−^) ions [4]. Recently, fluorapatite (FA), which is produced via the ionic substitution of HA with F^−^ ions, is more often used for bone regeneration and dental applications. It was demonstrated that FA stimulates osteoblast adhesion, proliferation, and the bone mineralization process [5,6].

Biphasic calcium phosphate (BCP), which is a combination of hydroxyapatite and β-tricalcium phosphate (β-TCP), is a synthetic material that is known for its osteoconduction, osteoinduction, and cell-mediated resorption properties, which make it an attractive option for bone regeneration in dental and orthopedic applications. Some research highlights the potential benefits of using β-TCP as a bone graft substitute and carrier for local administration of drugs. However, the studies also pointed out that β-TCP can exhibit inconsistent biological behavior. This suggests that more research is needed to fully understand the factors that influence the performance of β-TCP in bone regeneration. This is an important area of research that could have significant implications for the field of bone regeneration and orthopedics [7].

To improve surgical handiness and the mechanical parameters of bioceramics used for bone regeneration, biomaterials are frequently produced as composite materials made of a mineral phase (e.g., HA, FA, and TCP) and polymeric phase. The organic phase may be of natural (e.g., chitosan, agarose, alginate, collagen, and hyaluronic acid) or synthetic (e.g., poly (glycolic acid) (PGA), polylactic acid (PLA), polycaprolactone (PCL), and polyether ether ketone (PEEK)) origin [8]. Recently, curdlan, which is a linear bacterial 1,3-β-d-glucan with its unique ability to form a gel after the heating of its aqueous suspension, has been frequently used as a binder for bioceramics during the production of various bone implants and scaffolds [4,9].

Host defense peptides (HDPs) often called antimicrobial peptides (AMPs) are cationic peptides exhibiting various antimicrobial and immunomodulatory activities; due to their ability to stimulate the crosstalk between immune cells they can also promote healing [10]. Such activities of AMPs involve the modulation of some signaling pathways during inflammation, T-cell responses, the differentiation and polarization of macrophages and dendritic cells, wound repair, and the apoptosis of some immune cells [11]. Currently, some AMPs are being evaluated in preclinical studies; for example, magainin derived from frog skin has entered phase III of a clinical trial [12].

The natural mixture of peptides named antimicrobial porcine extract (AMPNE) is prepared from fresh porcine blood consisting of different AMPs, such as protegrins, PR-39, and prophenins [13]. This product apart from its confirmed antimicrobial activity plays an important immunomodulatory role [14,15,16]. Based on previous research, AMPNE appeared to be useful in the regulation of the inflammatory response, especially in the presence of or after previous stimulation with biomaterials [17,18]. Therefore, after obtaining promising results from porcine cells we decided to expand the scope of the research to human cells. As mentioned previously, the excessive stimulation of immune cells results in the higher secretion of pro-inflammatory mediators that may disturb the repair process. For this reason, the new tissue regeneration strategies, increasingly focusing on the immunomodulatory properties of different biomaterials and blood-derived products for tissue regeneration, draw increasing attention. According to some authors, different AMPs in the regulation of inflammation should be considered rather as regulators of immune homeostasis than simply as pro-inflammatory or anti-inflammatory factors [15]. Therefore, AMPNE was applied for the stimulation of neutrophil (MDM) cultures during incubation with different biomaterials to assess their effect on homologous (porcine) and heterologous (human) leukocytes.

We hypothesize that the physicochemical properties of biomaterials, such as surface topography, charge, and degradation rate, significantly influence the magnitude and nature of the immune response elicited. We also postulate that the immune response to biomaterials evolves over time, with distinct phases of inflammation, tissue remodeling, and potential immune tolerance induction. The primary objective of this study is to comprehensively examine the immune responses triggered by different biomaterials upon implantation in animal models. By characterizing the key immunological events at the implantation site, we aim to identify factors that contribute to either favorable integration or adverse reactions to biomaterials.

Among the various biomaterials for bone regeneration, calcium phosphates (CaP’s), including hydroxyapatite and its variants modified by ionic substitutions, α-tricalcium phosphate, and β-tricalcium phosphate, as well as CaP-based composite biomaterials, are most often used to fill bone defects. CaP biomaterials and their composites are characterized by high biocompatibility, osteoconductivity, and the ability to form apatite crystals after immersion in a simulated body fluid (bioactivity), ensuring good osseointegration after implantation. Thus, the first aim of this study was to evaluate the response of porcine neutrophils to two commercially available biomaterials belonging to CaP’s, namely hydroxyapatite (HA) and a mixture of hydroxyapatite and β-TCP (HATCP), and then to evaluate how AMPNE influences this response. Our second aim was to assess the effect of AMPNE as a form of heterologous stimulation of the activity of neutrophils and MDM after contact with some biomaterials. In this study, our focus lies in exploring the wide-ranging applications, diverse functionalities, and distinct immunological response mechanisms of HA, a HA-based composite (HAP), a fluoroapatite-based composite (FAP), and the polymeric curdlan matrix. These biomaterials have been specifically chosen due to their broad applicability and unique characteristics, which were discussed in the introduction. HAP and FAP composites (containing CaP’s and the chitosan/agarose polymeric matrix) were selected for this research as model composite biomaterials due to their previously proven high biocompatibility and optimal microstructural properties for a good osseointegration process. Since it is known that curdlan solution has the ability to activate macrophages and induce inflammatory reactions [19,20,21], the goal of this study was also to check whether or not curdlan suspension after heat-induced gelation process retains its pro-inflammatory properties. It should be noted that thermally gelled curdlan more often acts as a binder for bioceramics during biomaterial production [4,9]. It is also the component of a commercially available bone implant called FlexiOss^®^ (Medical Inventi S.A., Lublin, Poland). Therefore, by investigating varying functionalities of mentioned biomaterials and the different ways in which they generate immunological responses, we aim to gain a comprehensive understanding of these biomaterials. This research will contribute to expanding our knowledge of their potential applications in various fields and shed light on their promising properties for future advancements. These findings will add new data to the knowledge about the effect of a homologous and heterologous mixture of antimicrobial peptides on the activity of leukocytes in vitro as potential modifiers of the inflammatory response, which will be useful in preventing undesirable effects during biomaterial implantation.

In light of growing drug resistance, the preparation of a substance of biological origin containing a mixture of peptides with proven antimicrobial and immunomodulatory properties will be an important step in the development of biomaterial sciences and may be considered a therapeutic alternative to be used after clinical trials.

## 2. Materials and Methods

### 2.1. Biomaterials Used for the Study

Commercially available hydroxyapatite (HA BIOCER, Chema Elekromet Spółdzielnia Pracy, Rzeszów, Poland) and BCP, i.e., a mixture of hydroxyapatite and β-tricalcium phosphate (60% HA + 40% β-TCP, HT BIOCER, Chema Elekromet Spółdzielnia Pracy, Rzeszów, Poland) were used for the stimulation of porcine neutrophils in vitro. Before the experiment, biomaterials were suspended in sterile PBS on 24-wells plates at 37 °C with 5% CO_2_ for 2 h. Commercially available hydroxyapatite is referred to as HA, while BCP is referred to as HATCP throughout the manuscript.

A hydroxyapatite-based composite (referred to as HAP) and fluoroapatite-based material (referred to as FAP) were produced according to Polish Patent no. 235822. A detailed description of the fabrication method can be found in [22]. Briefly, biomaterials were made of a polymeric chitosan–agarose phase (both biopolymers were purchased from Sigma-Aldrich Chemicals, Warsaw, Poland) and mineral phase in the form of either hydroxyapatite nanopowder (Sigma-Aldrich Chemicals, Warsaw, Poland) or fluoroapatite nanopowder. Fluoroapatite was produced according to the procedure described previously [4]. The mixture of 2% (*w*/*v*) chitosan (50–190 kDa molecular weight), 5% (*w*/*v*) agarose (gel point 36 ± 1.5 °C) and 40% (*w*/*v*) bioceramics (HA or FA nanopowder) were prepared in 2% (*v*/*v*) acetic acid solution (Avantor Performance Materials, Gliwice, Poland). Then, a gas-foaming agent in the form of 2% (*w*/*v*) sodium bicarbonate (Sigma-Aldrich Chemicals, Warsaw, Poland) was added and the resultant mixture was subjected to heating at 95 °C in a water bath. Afterwards, the samples were cooled, frozen by placing them in a liquid-nitrogen vapor phase, freeze-dried, neutralized in 1% (*w*/*v*) sodium hydroxide solution (Avantor Performance Materials, Gliwice, Poland), washed with deionized water, and air-dried.

A curdlan matrix was prepared using curdlan powder purchased from Wako Chemicals (Osaka, Japan). Briefly, an 8% *w*/*v* curdlan suspension was prepared in deionized water and spread on the 13 mm diameter glass coverslip. Then, the glass coverslip was subjected to heating at 90 °C for 20 min to obtain thin thermally gelled curdlan samples.

### 2.2. Preparation of AMPNE

**Figure 1:**
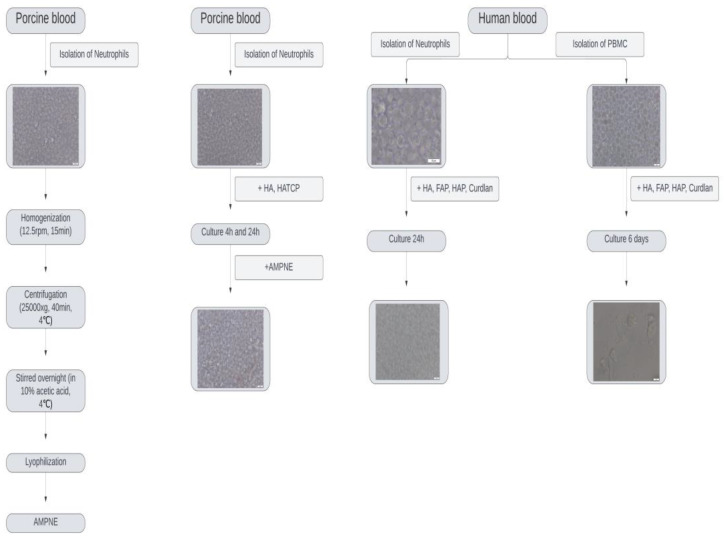
Schematic diagram of the study design.

Neutrophils were isolated from the porcine blood collected via slaughter according to the previously described method [13]. Before the experiment, the number and the viability of cells were evaluated using R1 Automated Cell Counter (Olympus, Warsaw, Poland). The purity of isolated cells was confirmed after Giemsa–May–Grunewald staining (85% neutrophils). The isolated neutrophils were disrupted using homogenizer DIAX 900 (Heidolph, Schwabach, Germany; 12.5 rpm for 15 min) and released granules were collected via centrifugation at 25.000× *g*, 40 min, 4 °C and stirred overnight in 10% acetic acid at 4 °C. The extract containing the antimicrobial peptides was separated from the granules (25.000× *g*, 20 min, 4 °C), divided into portions with a spectrophotometrically confirmed peptide concentration and lyophilized. The portions were stored at −70 °C until use (Figure 1).

### 2.3. Incubation of Porcine Neutrophils with HA or HATCP, with or without Stimulation with Homologous AMPNE

Neutrophil suspensions from porcine blood prepared as described above were seeded at a density of 1 × 10^6^ on 24-wells plates containing 25 mg of HA and HATCP previously prepared in a 500 μL PBS buffer and incubated at 37 °C with 5% CO_2_ for 4 h and 24. Thus, the ratio for each sample was the same and equal to 50 mg sample per 1 mL of the cell suspension. The representative cultures from all groups were additionally stimulated with 40 µg/mL of AMPNE and marked as HA + EXT and HATCP + EXT. Neutrophils without biomaterials were control group marked as N (Figure 1).

### 2.4. Isolation of Human Neutrophils and Preparation of Human Monocyte Derived Macrophages

Neutrophils were isolated from the human peripheral blood of healthy volunteers (informed consent was obtained from the healthy volunteers; approval was obtained from the Bioethics Committee of the Medical University of Lublin, no. KE-0254/187/10/2022) using the method described for the isolation of neutrophils from porcine blood.

Peripheral blood mononuclear cells (PBMC) were isolated from human whole blood (informed consent was obtained from the healthy volunteers; approval was obtained from the Bioethics Committee of the Medical University of Lublin no. KE-0254/187/10/2022) via Gradisol L (Aqua Medica, Janikowo, Poland) density gradient centrifugation as described previously for rabbit macrophages [23]. The suspension of PBMC in DMEM with 10% bovine calf serum (BCS) was adjusted to 1 × 10^6^ cells/mL and seeded in an amount of 500 μL onto the surfaces of the evaluated biomaterials (25 mg) placed in 24-well culture plates (the ratio was kept at 50 mg of the sample per 1 mL of the cell suspension), while 24-well plates without biomaterials served as controls (Figure 1).

### 2.5. Evaluation of the Response of Leukocytes to HA, HAP, FAP, or Curdlan; Stimulation of Cell Cultures with AMPNE

Human neutrophils were suspended in 500 μL of PBS at a density of 1 × 10^6^ and incubated at 37 °C with 5% CO_2_ for 24 h with different scaffolds (HA, HAP, and FAP) or with curdlan (25 mg of each sample was placed in the wells of a 24-well plate). The neutrophils tested without biomaterials comprised the control group, marked as N. Representative cultures from each group were additionally stimulated with neutrophils with 40 µg/mL of the heterologous extract. HA, HAP, FAP or curdlan and AMPNE were marked as HA + EXT, HAP + EXT, FAP + EXT, and N + C + EXT, respectively. Cultures of MDM were carried out analogically to those of human neutrophils; briefly the cells were incubated at 37 °C with 5% CO_2_ for 5 days (medium was changed after 24 h) and were marked as follows: MF as the control group without a biomaterial, HA, HAP, FAP, and curdlan, depending on the biomaterial used (25 mg of each sample per 500 μL of the cell suspension). After this period, the representative cultures from all groups were additionally stimulated with 40 µg/mL of AMPNE, cultured for 24 h under the same conditions and marked as the HA + EXT, HAP + EXT, FAP + EXT and curdlan groups, respectively. The groups without AMPNE were also incubated for an additional 24 h under the same conditions. After total incubation for 6 days, all described groups were evaluated with respect to the generation of ROS and RNS by MDM (Figure 1).

### 2.6. Assessment of Neutrophil and MDM Activity In Vitro

The measurements of the activity of porcine neutrophils in the presence of the tested materials were conducted after 4 h and 24 h of incubation. Enzyme release from azurophilic granules was assessed on the basis of elastase and MPO activity measured based on the cleavage of azocasein (Sigma-Aldrich) using a microplate reader, BioTek EL800 (BioTek, Janki, Poland). Alkaline phosphatase (ALP) release from secretory granules was determined after 10 min of incubation at 25 °C with the substrate 4-nitrophenyl phosphate disodium salt hexahydrate (Sigma-Aldrich, Poznan, Poland). A similar assessment was conducted on neutrophils isolated from human blood.

The nitrite accumulation in the medium, as a marker of NO production by porcine and human leukocytes, was tested using the Griess reaction and a standard curve of different concentrations of nitrite. In cultures of MDM, the generation of superoxide was measured as described previously. Briefly, after 15 min of incubation of the neutrophil or MDM cultures with 0.1% nitroblue tetrazolium solution (NBT, Sigma-Aldrich) at room temperature absorbance was read at 545 nm, and the generation of superoxide was calculated using the extinction coefficient of NBT (21.1 nMol) [23].

### 2.7. Statistical Analysis

All tests were conducted for three independent samples. Data were analyzed using Statistica Poland software (Statistica 13.1, Dell, Poland) with a one-tailed paired Student’s *t*-test and shown as mean ± standard deviation (SD). Differences between experimental groups and the control were considered statistically significant at *p* < 0.05.

## 3. Results

### 3.1. Porcine Neutrophil Activity in Contact with HA and HATCP, Effect of Homologous AMPNE

**Figure 2:**
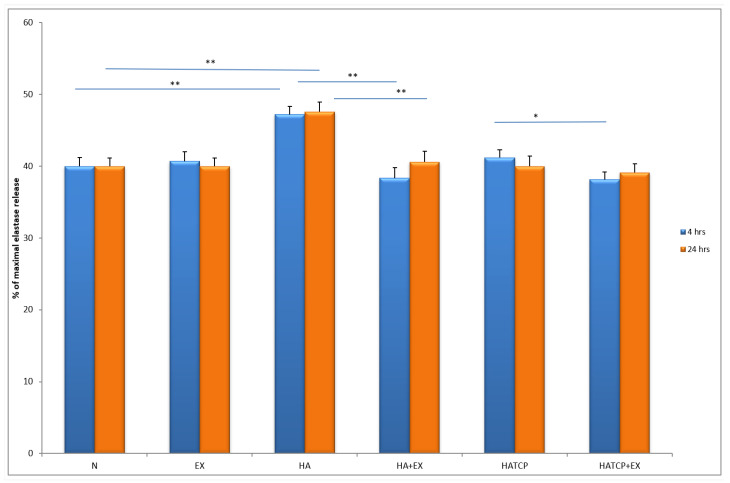
The % of maximal elastase release. N—porcine neutrophils; EX—porcine neutrophils + AMPNE isolated from porcine neutrophils; HA—porcine neutrophils + hydroxyapatite; HA + EX—porcine neutrophils + hydroxyapatite + AMPNE isolated from porcine neutrophils; HATCP—porcine neutrophils + hydroxyapatite with TCP (β-tricalcium phosphate); HATCP + EX—porcine neutrophils + hydroxyapatite with TCP (β-tricalcium phosphate) + AMPNE isolated from porcine neutrophils. Data were acquired in triplicates, analyzed with a one-tailed paired Student’s *t*-test, and presented with the mean ± SD. * *p* < 0.05; ** *p* < 0.01.

**Figure 3:**
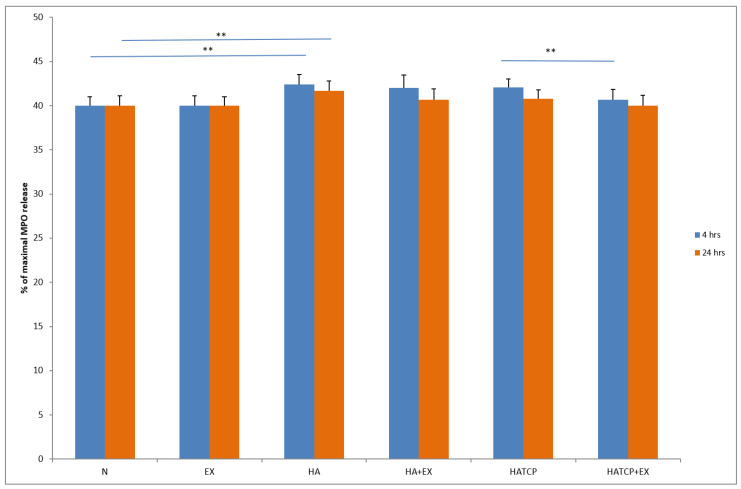
The % of MPO release. N—porcine neutrophils; EX—porcine neutrophils + AMPNE isolated from porcine neutrophils; HA—porcine neutrophils + hydroxyapatite; HA + EX—porcine neutrophils + hydroxyapatite + AMPNE isolated from porcine neutrophils; HATCP—porcine neutrophils + hydroxyapatite with TCP (β-tricalcium phosphate); HATCP + EX—porcine neutrophils + hydroxyapatite with TCP (β-tricalcium phosphate) + AMPNE isolated from porcine neutrophils. Data were acquired in triplicates, analyzed with a one-tailed paired Student’s *t*-test, and presented with the mean ± SD. ** *p* < 0.01.

**Figure 4:**
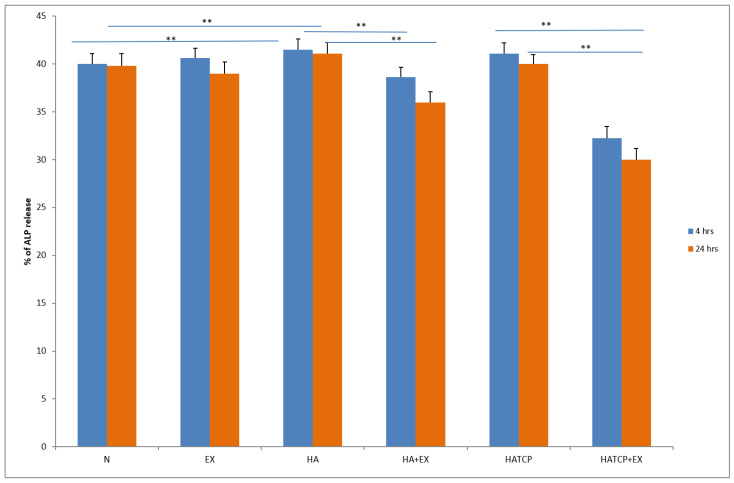
The % of ALP release. N—porcine neutrophils; EX—porcine neutrophils + AMPNE isolated from porcine neutrophils; HA—porcine neutrophils + hydroxyapatite; HA + EX—porcine neutrophils + hydroxyapatite + AMPNE isolated from porcine neutrophils; HATCP—porcine neutrophils + hydroxyapatite with TCP (β-tricalcium phosphate); HATCP + EX—porcine neutrophils + hydroxyapatite with TCP (β-tricalcium phosphate) + AMPNE isolated from porcine neutrophils. Data were acquired in triplicates, analyzed with a one-tailed paired Student’s *t*-test, and presented with the mean ± SD. ** *p* < 0.01.

**Figure 5:**
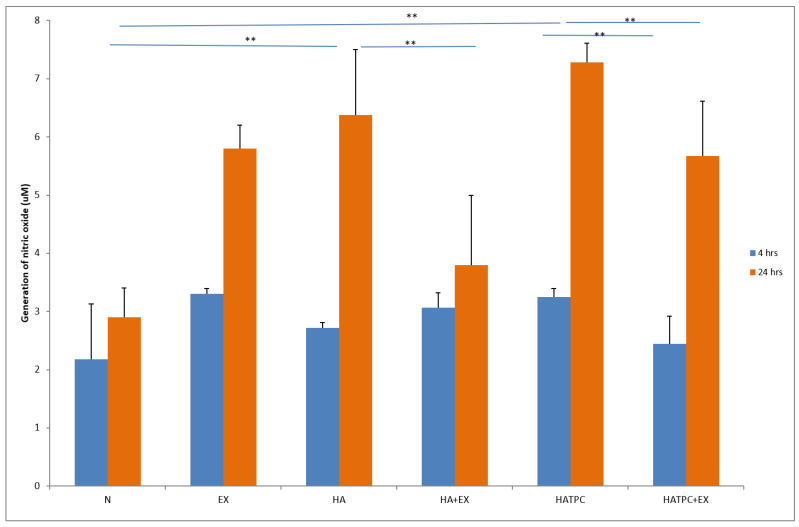
Generation of nitric oxide (uM). N—porcine neutrophils; EX—porcine neutrophils + AMPNE isolated from porcine neutrophils; HA—porcine neutrophils + hydroxyapatite; HA + EX—porcine neutrophils + hydroxyapatite + AMPNE isolated from porcine neutrophils; HATCP—porcine neutrophils + hydroxyapatite with TCP (β-tricalcium phosphate); HATCP + EX—porcine neutrophils + hydroxyapatite with TCP (β-tricalcium phosphate) + AMPNE isolated from porcine neutrophils. Data were acquired in triplicates, analyzed with a one-tailed paired Student’s *t*-test, and presented with the mean ± SD. ** *p* < 0.01.

The activity of neutrophils estimated based on enzyme release from primary granules, namely elastase and MPO, increased significantly (*p* < 0.05) in cultures with HA at both time points, after 4 h and 24 h of incubation, compared to unstimulated culture. The response of neutrophils to HATCP was less pronounced. The response from secretory granules based on ALP revealed that stimulation with HA generated an increased release of ALP in comparison with the unstimulated control. The response of neutrophil secretory granules to HATCP was less pronounced. The additional stimulation of neutrophil cultures with AMPNE caused a decrease in enzymatic response. Elastase, MPO and ALP release was significantly inhibited by treatment with AMPNE at both time points in the HA cultures, and to a lesser extent in the HATCP cultures (Figure 2, Figure 3 and Figure 4). NO generation significantly increased in groups HA and HATCP after 24 h of incubation in comparison to that in the unstimulated neutrophil culture. The inhibitory effect of AMPNE was evident in all cases (Figure 5).

### 3.2. Human Neutrophil Activity after Incubation with HA, HAP, FAP, or Curdlan; the Effect of Heterologous AMPNE

**Figure 6:**
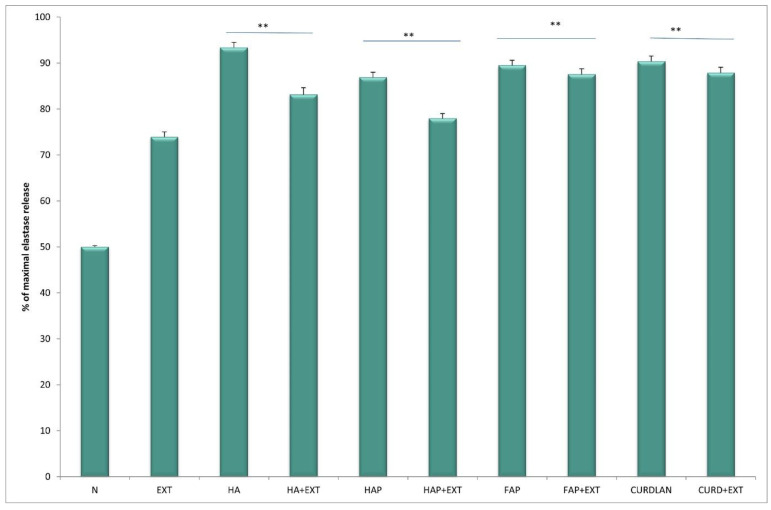
The % of maximal elastase release. N—human neutrophils; EXT—human neutrophils + AMPNE isolated from porcine neutrophils; HA—human neutrophils + hydroxyapatite; HA + EXT—human neutrophils + hydroxyapatite + AMPNE isolated from porcine neutrophils; HAP—human neutrophils + HAP; HAP + EXT—human neutrophils + HAP + AMPNE isolated from porcine neutrophils; FAP—human neutrophils + FAP; FAP + EXT—human neutrophils + FAP + AMPNE isolated from porcine neutrophils; CURDLAN—human neutrophils + CURDLAN; CURD + EXT—human neutrophils + CURDLAN + AMPNE isolated from porcine neutrophils. Data were acquired in triplicates, analyzed with a one-tailed paired Student’s *t*-test, and presented with the mean ± SD. ** *p* < 0.01.

**Figure 7:**
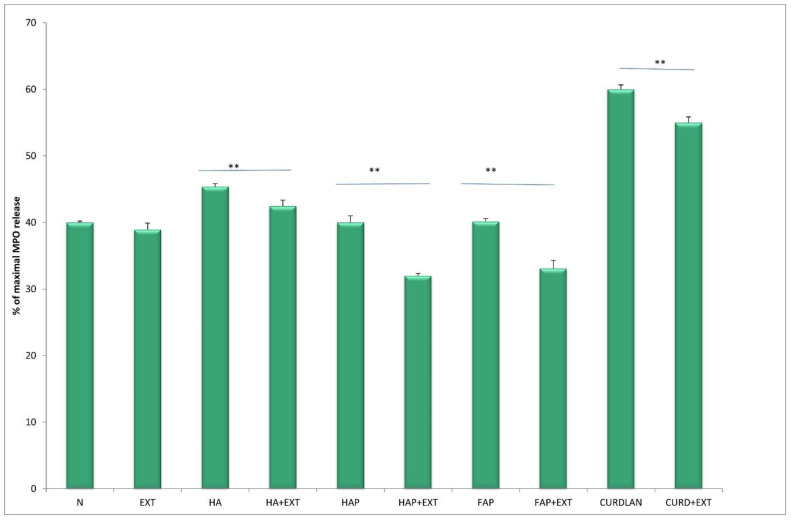
The % of maximal MPO release. N—human neutrophils; EXT—human neutrophils + AMPNE isolated from porcine neutrophils; HA—human neutrophils + hydroxyapatite; HA + EXT—human neutrophils + hydroxyapatite + AMPNE isolated from porcine neutrophils; HAP—human neutrophils + HAP; HAP + EXT—human neutrophils + HAP + AMPNE isolated from porcine neutrophils; FAP—human neutrophils + FAP; FAP + EXT—human neutrophils + FAP + AMPNE isolated from porcine neutrophils; CURDLAN—human neutrophils + CURDLAN; CURD + EXT—human neutrophils + CURDLAN + AMPNE isolated from porcine neutrophils. Data were acquired in triplicates, analyzed with a one-tailed paired Student’s *t*-test, and presented with the mean ± SD. ** *p* < 0.01.

**Figure 8:**
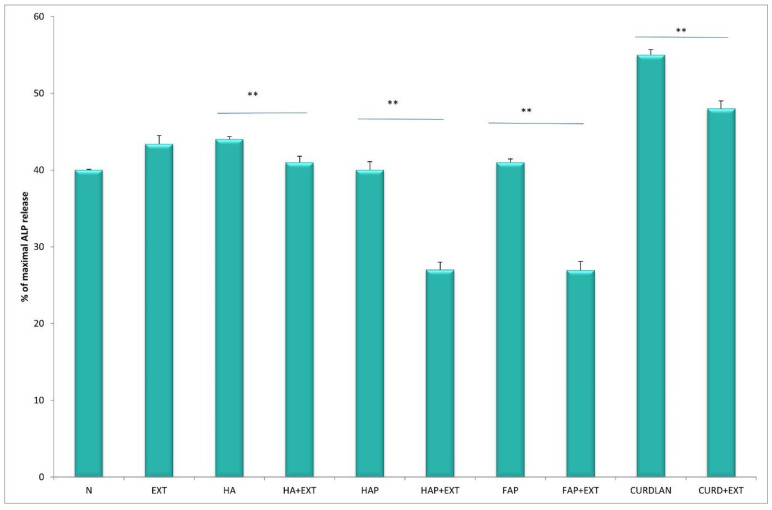
The % of maximal ALP release. N—human neutrophils; EXT—human neutrophils + AMPNE isolated from porcine neutrophils; HA—human neutrophils + hydroxyapatite; HA + EXT—human neutrophils + hydroxyapatite + AMPNE isolated from porcine neutrophils; HAP—human neutrophils + HAP; HAP + EXT—human neutrophils + HAP + AMPNE isolated from porcine neutrophils; FAP—human neutrophils + FAP; FAP + EXT—human neutrophils + FAP + AMPNE isolated from porcine neutrophils; CURDLAN—human neutrophils + CURDLAN; CURD + EXT—human neutrophils + CURDLAN + AMPNE isolated from porcine neutrophils. Data were conducted in triplicates, analyzed with a one-tailed paired Student’s *t*-test, and presented with the mean ± SD. ** *p* < 0.01.

**Figure 9:**
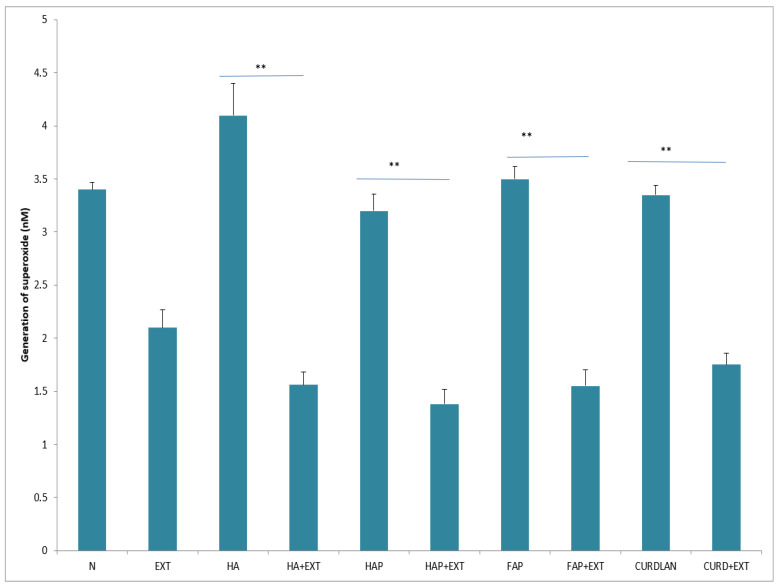
Generation of superoxide. N—human neutrophils; EXT—human neutrophils + AMPNE isolated from porcine neutrophils; HA—human neutrophils + hydroxyapatite; HA + EXT—human neutrophils + hydroxyapatite + AMPNE isolated from porcine neutrophils; HAP—human neutrophils + HAP; HAP + EXT—human neutrophils + HAP + AMPNE isolated from porcine neutrophils; FAP—human neutrophils + FAP; FAP + EXT—human neutrophils + FAP + AMPNE isolated from porcine neutrophils; CURDLAN—human neutrophils + CURDLAN; CURD + EXT—human neutrophils + CURDLAN + AMPNE isolated from porcine neutrophils. Data were acquired in triplicates, analyzed with a one-tailed paired Student’s *t*-test, and presented with the mean ± SD. ** *p* < 0.01.

**Figure 10:**
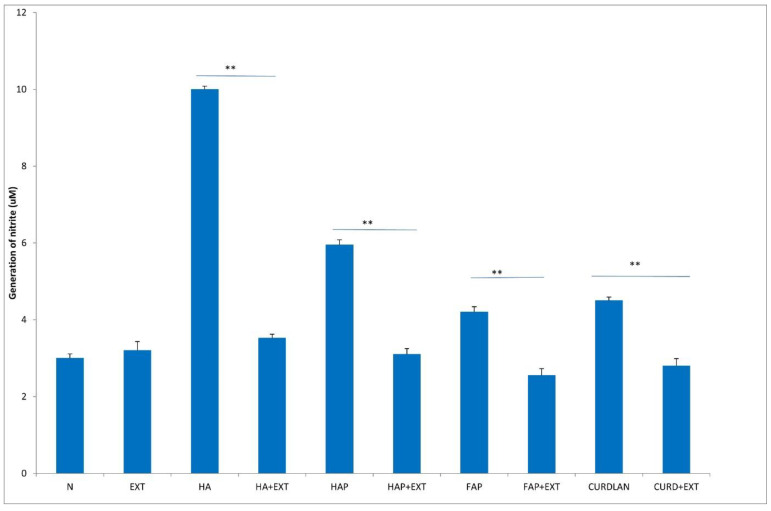
Generation of nitrite. N—human neutrophils; EXT—human neutrophils + AMPNE isolated from porcine neutrophils; HA—human neutrophils + hydroxyapatite; HA + EXT—human neutrophils + hydroxyapatite + AMPNE isolated from porcine neutrophils; HAP—human neutrophils + HAP; HAP + EXT—human neutrophils + HAP + AMPNE isolated from porcine neutrophils; FAP—human neutrophils + FAP; FAP + EXT—human neutrophils + FAP + AMPNE isolated from porcine neutrophils; CURDLAN—human neutrophils + CURDLAN; CURD + EXT—human neutrophils + CURDLAN + AMPNE isolated from porcine neutrophils. Data were acquired in triplicates, analyzed with a one-tailed paired Student’s *t*-test, and presented with the mean ± SD. ** *p* < 0.01.

After incubation with HA, the enzymatic activity of neutrophils in with respect to elastase, MPO and ALP release increased, whereas the addition of AMPNE reduced this activity (Figure 6, Figure 7 and Figure 8). Neutrophils cultured with HA generated higher amounts of superoxide than did the control, Whereas HAP, FAP, or curdlan generated amounts of superoxide similar to those of neutrophils from the unstimulated culture. In all cases, neutrophil activity was significantly diminished by the addition of AMPNE (Figure 9). We observed a significant increase in NO generation (*p* < 0.05) after contact with the biomaterials tested and a significant decrease in this response after treatment with AMPNE. The highest response was noted in the group of the HA scaffold (10.00 ± 0.08 vs. 3.0 ± 0.11 µM); in other groups NO generation was also significantly higher than in the control group (Figure 10).

### 3.3. Human Macrophage Response to Biomaterials, Effect of Heterologous AMPNE

**Figure 11:**
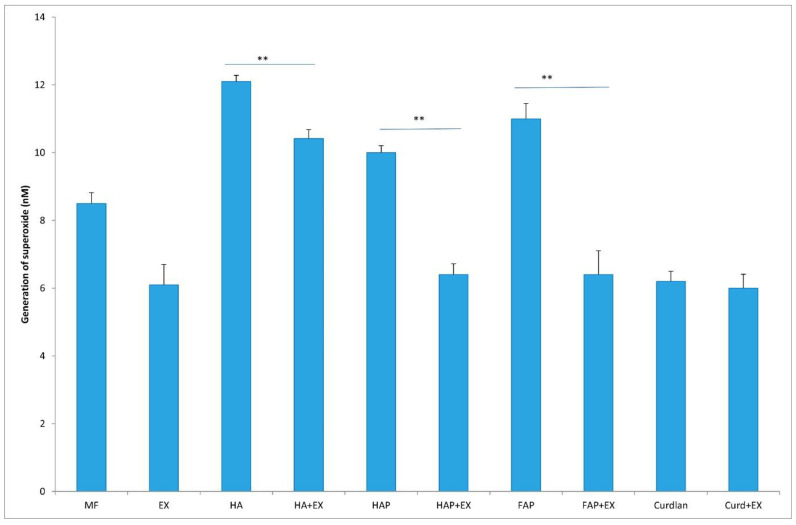
Generation of superoxide. MF—human macrophages; EX—human macrophages + AMPNE isolated from porcine neutrophils; HA—human macrophages + hydroxyapatite; HA + EX—human macrophages + hydroxyapatite + AMPNE isolated from porcine neutrophils; HAP—human macrophages + HAP; HAP + EX—human macrophages + HAP + AMPNE isolated from porcine neutrophils; FAP—human macrophages + FAP; FAP + EX—human macrophages + FAP + AMPNE isolated from porcine neutrophils; Curdlan—human macrophages + CURDLAN; Curd + EX—human macrophages + CURDLAN + AMPNE isolated from porcine neutrophils. Data were conducted in triplicates, analyzed with one-tailed paired Student’s *t*-test, and presented with the mean ± SD. ** *p* < 0.01.

**Figure 12:**
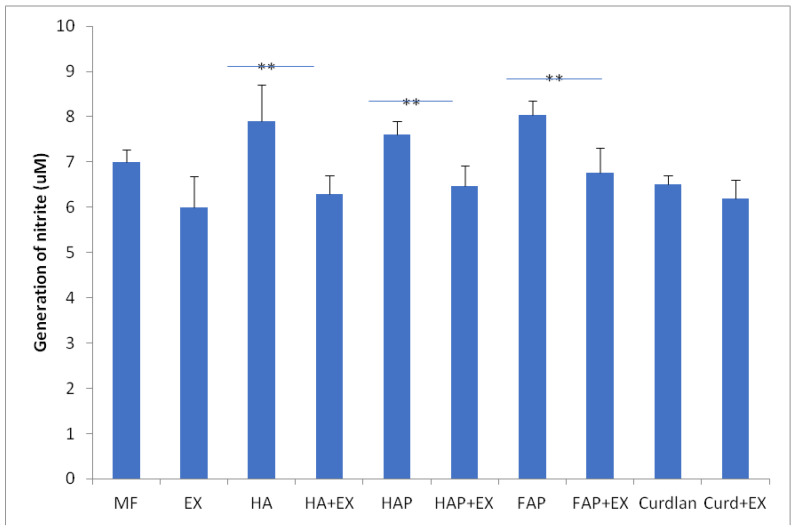
Generation of nitrite. MF—human macrophages; EX—human macrophages + AMPNE isolated from porcine neutrophils; HA—human macrophages + hydroxyapatite; HA + EX—human macrophages + hydroxyapatite + AMPNE isolated from porcine neutrophils; HAP—human macrophages + HAP; HAP + EX—human macrophages + HAP + AMPNE isolated from porcine neutrophils; FAP—human macrophages + FAP; FAP + EX—human macrophages + FAP + AMPNE isolated from porcine neutrophils; Curdlan—human macrophages + CURDLAN; Curd + EX—human macrophages + CURDLAN + AMPNE isolated from porcine neutrophils. Data were aquired in triplicates, analyzed with a one-tailed paired Student’s *t*-test, and presented with the mean ± SD. ** *p* < 0.01.

Macrophages cultured with HA shown higher generation of superoxide and NO than macrophages cultured in DMEM + BCS as control group without stimulation with biomaterial. Contrary, contact with curdlan caused decrease in macrophages activity compared to control group. In groups of HA, HAP and FAP stimulation with AMPNE caused significant decrease in superoxide and NO generation. This effect was not observed in curdlan group (Figure 11 and Figure 12).

## 4. Discussion

The main goal of this study was an evaluation of the response of two subpopulations of leukocytes, namely neutrophils and MDM, to some biomaterials, and the potential effect of AMPNE on this cellular response. Excessive neutrophil activity at sites of implantation can cause damage to surrounding tissues with prolonged inflammation and/or impaired wound healing. Therefore, the modulation of neutrophil activity appeared crucial for tissue regeneration. The recruited neutrophils could interact with the implanted biomaterial directly via binding sites on the material surface or indirectly through the surface-absorbed proteins, thus triggering dynamic interactions between the material and the inflammatory cells [1]. We evaluated the in vitro neutrophil response to some biomaterials based on enzyme release and RNS generation. During the first step of the experiment, we stated that porcine neutrophils enhanced their activity after contact with HA. Previous studies have shown that cellular responses can be triggered by the presence of spontaneously formed calcium phosphate particles or by the presence of complexes formed between calcium phosphate particles and proteins resulting in particle internalization by host cells and the subsequent activation of different pro-inflammatory responses. Nonetheless, it remains unclear whether or not the particles could interact with neutrophils, or how the inflammatory response may be propagated [24]. In the experiment of Peng et al. the influence of mineral particles on neutrophil activity was assessed after two hours of incubation. We extended the scope of the research by measuring the neutrophil response after 4 and 24 h of incubation. Circulating neutrophils usually have been regarded as short-living cells with a 6–8 h half-life, whereas for activated neutrophils during inflammation their life span increases several times [1,25]. Therefore, in the light of fact that neutrophils are no longer considered only as short-living cells and that their involvement in inflammation is more long-lasting and comprehensive, our experiment was continued for 24 h.

Since the porcine model is relevant for regenerative medicine and tissue engineering, as well as biomechanics studies, and the obtained results have translational potential for humans, we decided to start our research on porcine blood components [26,27].

This study revealed that after contact with HA β-TCP, porcine neutrophils did not release significantly higher amounts of enzymes at both time points compared to unstimulated cultures, whereas NO generation was higher in cultures incubated for 24 h. These results were consistent with previous reports describing CaP ceramics, such as β-TCP as stimulators of the inflammatory cell response, when tested as particles or following the resorption of calcium-containing biomaterials [2,3].

In the second step of the experiment, the response of human neutrophils to different biomaterials was evaluated in vitro. Human neutrophils after incubation with HA released higher amounts of granule enzymes and generated higher amounts of superoxide and NO than did control neutrophils. To our knowledge, the enzymatic response of neutrophils to some studied materials (HAP, FAP, and curdlan) has not been evaluated to date. However, the increased enzymatic response to HA together with elevated NO generation by human neutrophils was observed previously by Velard et al. These authors estimated that following biomaterial implantation, released HA particles activated human neutrophils toward the increased production of pro-inflammatory cytokines such as, IL-1a and IL-8. In addition, other works showed that the implantation of HA caused an early activation of the innate immune response [1]. Similarly, neutrophils after incubation with curdlan released higher amounts of enzymes, superoxide and NO than did control neutrophils. The increased activity of neutrophils after stimulation with curdlan was previously noted by some authors [28]. Contact with HAP or FAP did not evoke a significant neutrophil response. A previous report indicated that HAP and FAP did not generate a serious pro-inflammatory response from immune cells in vitro [4].

Macrophages cultured with HA generated higher amounts of superoxide and NO than did macrophages cultured only with DMEM + BCS, whereas changes in macrophage activity under the influence of HAP and FAP were insignificant. Previous reports have suggested that HA can produce particles that can be recognized as a foreign material and initiate not only the activation of neutrophils but also the recruitment and activation of macrophages. The resulting excessive cellular response can eventually result in chronic inflammation, fibrosis, and a further destruction of surrounding tissues [29]. However, it should be considered that macrophages are heterogonous and highly plastic cells that undergo different forms of activation depending on the signaling stimuli from the local microenvironment. Their activation and/or polarization may be caused by many different factors involved in the properties of studied materials [1,29,30]. Therefore, we encountered a different response to HAP and FAP. On the other hand, curdlan caused a decrease in macrophage activity compared to control group. Some authors reported the inhibition of a pro-inflammatory response to β-glucan [31]. Additionally, Bekkering et al. observed a decreased ROS generation in macrophages after stimulation with β-glucan [16].

Based on the conducted research, it can be concluded that the tested biomaterials affect neutrophils and macrophages in different ways, increasing or decreasing their pro-inflammatory activity. Traditionally, the immunomodulatory effects of biomaterials on leukocytes center the decrease in their pro-inflammatory activation to alleviate healing disturbances or incomplete tissue integration caused by the excessive inflammatory response [1]. However, novel immuno-informed biomaterials can be considered in two categories. The first group is composed of replacement biomaterials that integrate with tissue and remain stable on implantation site; these materials cause minimal inflammation and fibrous tissue formation.

The second group is formed of the biomaterials for regenerative medicine that enhance tissue formation during their sustained and controlled degradation.

Our interest was in the second group of biomaterials to modulate an excessive undesired inflammatory response by using AMPNE. Decisions about the choice of the biomaterial should be based on their properties and possible interaction with host immune cells to limit the possible inflammatory response, to prevent excessive inflammation and to enhance tissue regeneration [32]. Thus, our hypothesis was that AMPNE could a be useful blood-derived product for modulating the local inflammatory reaction and to suppress its excessive signs in order to prevent implant rejection. The development of factors modulating different leukocyte responses directed towards increased reparative properties can be an intriguing challenge for regenerative medicine [1]. Previous results indicated that macrophage polarization especially can be changed in many ways, creating some potential applications for immuno-informed biomaterials and blood-derived products [32].

Therefore, one of main aims of this study was an evaluation of the role of AMPNE in the modulation of leukocyte responses to tested biomaterials as a measure to diminish the excessive host response to biomaterials. The obtained results pointed out that the stimulation of neutrophils with homologous AMPNE decreased their secretory activity. Previously, the autologous inhibitory effect on secretory activity of neutrophils from other species was confirmed [23]. In the next part of the experiment, the heterologous effect of AMPNE on cultures of human neutrophils and MDM was evaluated in the presence of different biomaterials. Similar to the homologous application of AMPNE, after the addition of AMPNE to human neutrophils, a decrease in activity was observed under the influence of HA, HAP and FAP or curdlan. To our knowledge, any previous experiment has been performed with the application of porcine AMPs to human neutrophils seeded on the biomaterials studied. Neutrophil-derived extracts containing a mixture of different AMPs were initially used as antimicrobial natural products obtained from the blood of farm animals [33]. However, these blood-derived products have also exhibited immunomodulatory and tissue repair properties, enabling their wider application in regenerative medicine. The porcine neutrophil extract of antimicrobial peptides, prepared via isolation from neutrophil granules and lyophilized, is resistant to long-term storage and could be used as an autologous or heterologous product to modulate the inflammatory process and enhance healing. Some in vitro studies indicated that the addition of an AMP extract into neutrophil and macrophage cultures changed the activity of inflammatory cells obtained through biomaterial implantation in the animal model [23].

On the basis of previous research, AMPNE appeared to be promising candidate in regulation of inflammatory response, especially in the presence of or after previous stimulation with biomaterials [16,17]. Therefore, after obtaining promising results in porcine cells we decided to expand the scope of the research to human cells.

Neutrophil-derived extracts containing a mixture of different AMPs were initially used as antimicrobial natural products obtained from the blood of farm animals.

However, in addition to their well-known antimicrobial activity, these blood-derived products have also been proven to exhibit immunomodulatory and tissue repair properties, enabling their wider application in regenerative medicine. A crude neutrophil extract, obtained via isolation from neutrophil granules and lyophilized, is resistant to long-term storage and could be used as an autologous or heterologous product to promote the healing process in different tissues.

Neutrophil extracts differ in composition depending on the animal species. When obtained from isolated porcine neutrophils, they contain the largest variety of cathelicidins, including PR-39, prophenins, and protegrins. However, they lack defensins [15].

The stimulation of human macrophages cultured in the presence of HA, HAP, or FAP with AMPNE caused a significant decrease in ROS and RNS generation compared to cultures without AMPNE. This effect confirmed the previous findings that AMPs are immunomodulatory agents and compounds involved in the tissue repair process. It was established that human cathelicidin LL-37 could be a stimulator of the formation of new blood vessels and wound healing [34,35].

Murine cathelicidin-related antimicrobial peptide (CRAMP), like LL-37 in humans, also promotes wound repair. These properties of AMPs found their applications in regenerative medicine where human cathelicidin LL-37 was mainly used. A previous report described an AMP-loaded wound dressing with good biocompatibility, a controlled release of AMPs, and activity against different bacterial strains as a measure for the treatment of infected wounds. These wound dressings improved healing via the sustained release of the AMPs, which additionally modulated inflammatory cells, increased collagen deposition, and improved angiogenesis [12]. Another strategy is the use of AMPs in antimicrobial surface coatings on implants and other medical devices to prevent biofilm formation and the inflammatory response. For this purpose, the scaffold layer on the HA surface of titanium substrates was functionalized with LL-37 [36].

In the case of porcine AMPs, the observed anti-inflammatory effect of AMPNE on cell cultures seems to be related to one or more of its components. The most promising component is porcine cathelicidin PR-39 with its confirmed anti-inflammatory effect. Previous experiments on a murine model revealed that this cathelicidin is a potent angiogenic stimulator that blocks the ubiquitin proteasome-dependent degradation of hypoxia-inducible factor-1α protein to promote angiogenesis. Additionally, PR-39 can diminish NADPH oxidase-induced superoxide generation to decrease cell injury during inflammation and induce the expression of syndecans with the potential for tissue repair [37].

A limitation of this study is that was conducted in vitro and needs further preclinical trials. Moreover, a naturally derived mixture of different peptides needs to be evaluated in terms of active substances and their possible synergistic interaction which needs additional research to evaluate the molecular basis of their properties.

The further evaluation of the specific content of this extract is necessary; however, it should be underlined that the activity of particular components may be synergistic and the immunomodulatory effect may be strictly related to some components [38].

Considering all the obtained results from the studied leukocytes, macrophages appeared to be the most attractive targets for cell therapy because their phenotypes differ depending on the microenvironment and during cell therapy they can be replaced by the addition of generated or educated cells. Macrophage-based therapies seem to have fewer adverse effects and could be more reversible than other ones targeted at other cell populations. These experimental therapies could inhibit damaging microenvironmental signals during injury and/or enhance the pro-repair functions of stimulated macrophages [37,39,40].

## 5. Conclusions

These studies shed new light on host–implant interactions. First, the immunomodulatory properties of the tested biomaterials were confirmed based on neutrophils and the MDM response in vitro. Then, the response of cultured cells to homologous or heterologous stimulation with AMPNE was assessed. The addition of homologous or heterologous AMPNE to biomaterials causing an enhanced immune response suppressed this response both in neutrophils and macrophages. The obtained results confirmed their potential to reduce the excessive response of both neutrophils and MDM to the biomaterials, in order to protect against adverse effects after implant insertion. These properties, after clinical trials, may be used in preventing complications after orthopedic procedures related to the implantation of biomaterial in humans and animals. This study extends the knowledge on the hemocompatibility of biomaterials, in the research going beyond standard biocompatibility testing. The addition of AMPNE may be particularly useful in modulating the activity of macrophages during biomaterial implantation.

The ability of AMPNE to modulate the immune response during implantation opens up new possibilities for regenerative medicine approaches. By controlling the host response to biomaterials, it could enhance tissue regeneration and promote the better integration of implants with the host’s body. This could lead to improved outcomes in patients undergoing various regenerative therapies.

In tissue engineering, the success of engineered tissues largely depends on their interaction with the host environment. AMPNE’s immunomodulatory properties may be harnessed to create biomaterials that provoke minimal adverse reactions from the immune system and allow for better tissue integration. This could facilitate the development of more biocompatible and functional tissue-engineered constructs.

Excessive inflammation and immune reactions to implanted biomaterials can compromise their long-term stability and functionality. By using AMPNE to modulate macrophage activity and reduce host responses, the research may pave the way for more durable and reliable implants that maintain their performance over extended periods.

Implant-associated complications, such as infections, fibrosis, and rejection, are significant concerns in clinical settings. By using AMPNE as an immunomodulatory agent, these complications could potentially be mitigated, leading to improved patient outcomes and reducing the need for implant replacements or revisions.

It is essential to discuss the potential pathways for translating this research from the laboratory to clinical practice. Addressing challenges and outlining potential strategies for scaling up and optimizing the use of AMPNE in human trials will strengthen the practical relevance of this study.

## 6. Patents

The method of the production of apatite-based scaffolds is protected by Polish patent no. 235822.

## Data Availability

Data is contained within the article.
